# Supplementary material for: Identification of a small molecule that stimulates human β-cell proliferation and insulin secretion, and protects against cytotoxic stress in rat insulinoma cells
Source: PLoS One. 2020 Mar 16;15(3):e0224344. doi: 10.1371/journal.pone.0224344 (PMC7075568; doi:10.1371/journal.pone.0224344)
Supplement: S1 Fig — Rat islets were cultured for 72 h in the presence of various doses of GNF-9228 or DMSO (vehicle control). EdU was added for the last 18 h of culture. Islets were dispersed and stained for EdU incorporation. Immunofluorescent signals were detected and quantified with a Thermo Scientific Cellomics CX5 High Content (HC) cell imaging system. Data are expressed as mean +/- S.E.M. fold-increase in EdU positive cells compared to DMSO control from 3 independent rat islet preparations. In one set of rat islets, 20 μM GNF-9228 had no additional effect on rat islet cell EdU incorporation beyond the effect of the 10 μM dose. (PDF) [file pone.0224344.s001.pdf]

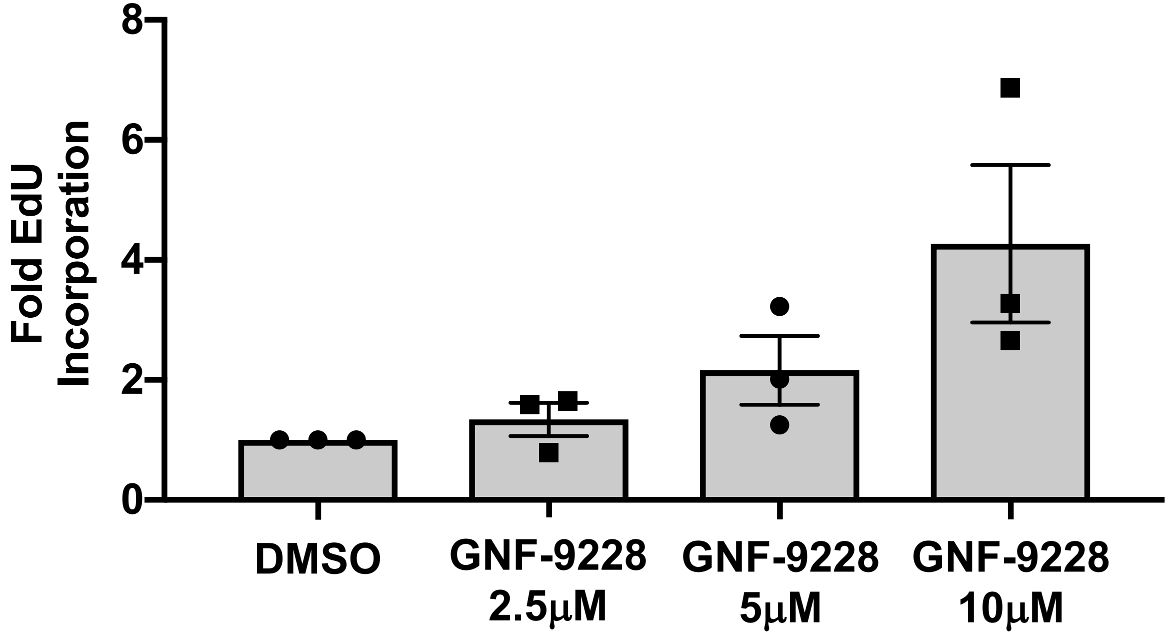

Supplemental Figure 1. Dose titration of GNF-9228 in rat islets.
Rat islets were cultured for 72 h in the presence of various doses of GNF-9228 or DMSO (vehicle control). EdU was added for the last 18 h of culture. Islets were dispersed and stained for EdU incorporation. Immunofluorescent signals were detected and quantified with a Thermo Scientific Cellomics CX5 High Content (HC) cell imaging system. Data are expressed as mean +/- S.E.M. fold-increase in EdU positive cells compared to DMSO control from 3 independent rat islet preparations. In one set of rat islets, 20 μM GNF-9228 had no additional effect on rat islet cell EdU incorporation beyond the effect of the 10 μM dose.
