# Supplementary material for: Identification of a small molecule that stimulates human β-cell proliferation and insulin secretion, and protects against cytotoxic stress in rat insulinoma cells
Source: PLoS One. 2020 Mar 16;15(3):e0224344. doi: 10.1371/journal.pone.0224344 (PMC7075568; doi:10.1371/journal.pone.0224344)
Supplement: S2 Fig — Human islets were treated with 16.7 mM glucose for 1 h in the presence of 10 μM GNF-9228 or DMSO. Data are from 3 islet preparations from independent donors, each assayed in quadruplicate, and are expressed as mean ± S.E.M. of insulin secreted at 16.7 mM glucose normalized to DMSO-treated cells. (PDF) [file pone.0224344.s002.pdf]

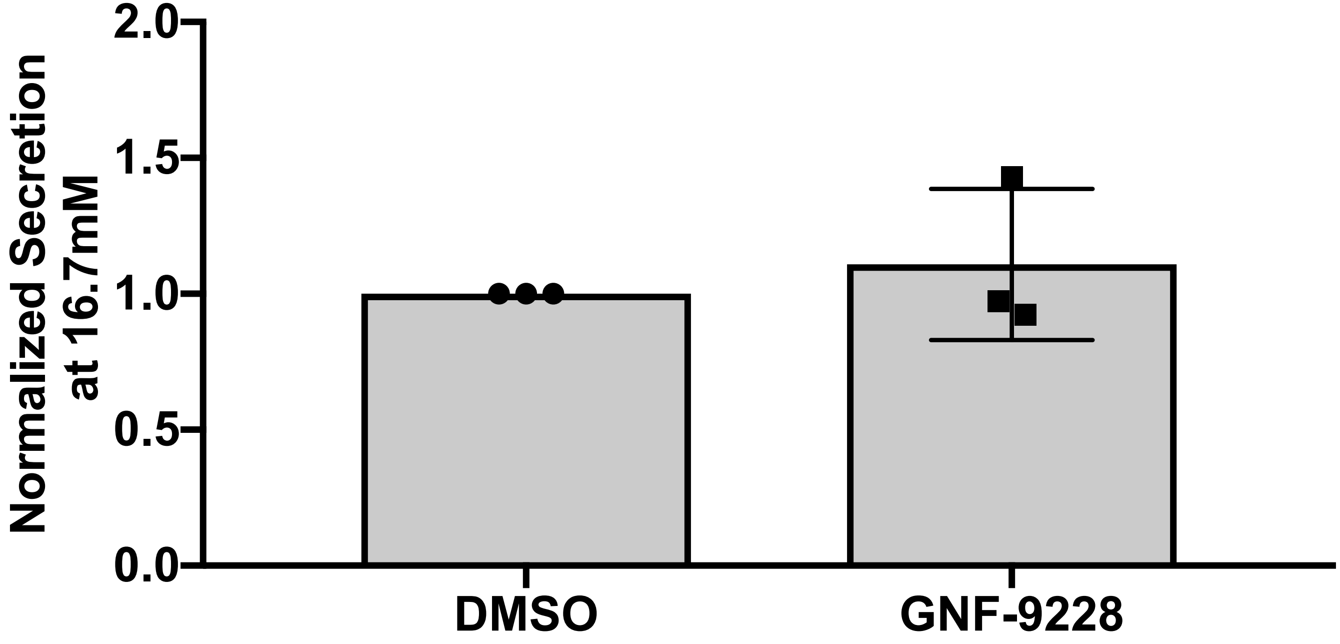

Supplemental Figure 2. Lack of acute effect of GNF-9228 on insulin secretion in human islets. Human islets were treated with 16.7 mM glucose for 1 h in the presence of 10 μM GNF-9228 or DMSO. Data are from 3 islet preparations from independent donors, each assayed in quadruplicate, and are expressed as mean ± S.E.M. of insulin secreted at 16.7 mM glucose normalized to DMSO-treated cells.
