# Supplementary material for: Identification of a small molecule that stimulates human β-cell proliferation and insulin secretion, and protects against cytotoxic stress in rat insulinoma cells
Source: PLoS One. 2020 Mar 16;15(3):e0224344. doi: 10.1371/journal.pone.0224344 (PMC7075568; doi:10.1371/journal.pone.0224344)
Supplement: S3 Fig — A preparation of human islets was cultured in the presence of 2.5μM, 5μM GNF-9228 or 10μM GNF-9228 for 72h and then subjected to glucose stimulated insulin secretion. (Data represent mean+ Std.Dev. measured in triplicate of 30 islets) (PDF) [file pone.0224344.s003.pdf]

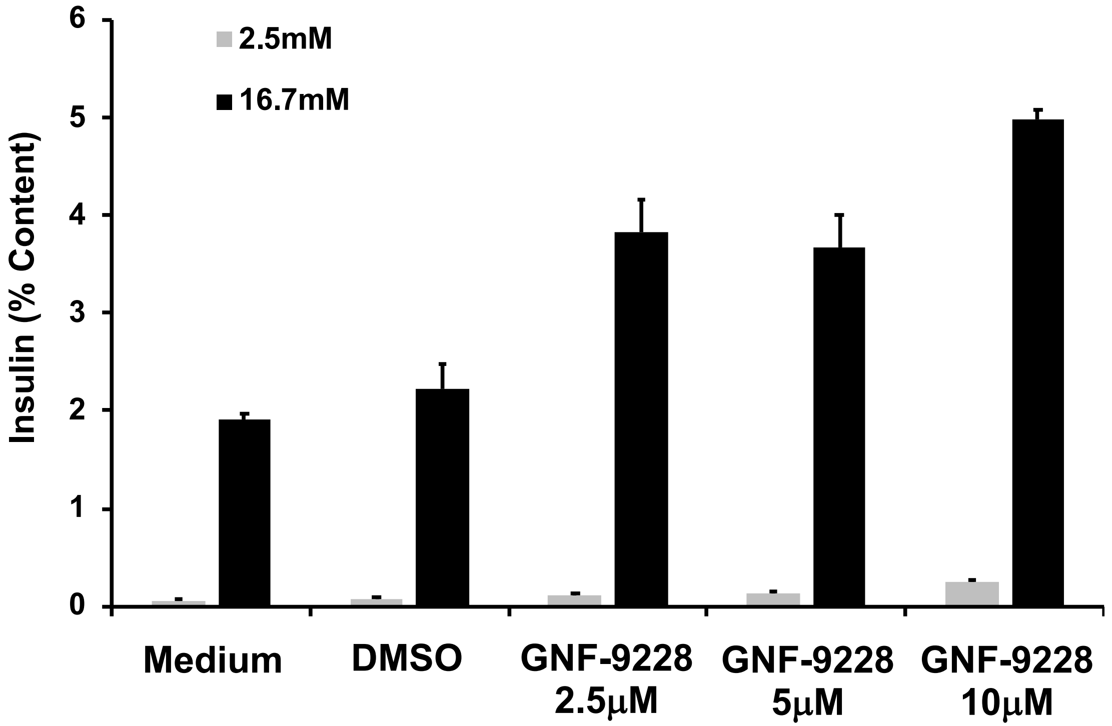

Supplemental Figure 3. Glucose stimulated insulin in human islets after 72h incubation with lower concentration of GNF-9228. A preparation of human islets was cultured in the presence of 2.5μM, 5μM GNF-9228 or 10μM GNF-9228 for 72h and then subjected to glucose stimulated insulin secretion. (Data represent mean+ Std.Dev. measured in triplicate of 30 islets)
