# Supplementary material for: Identification of a small molecule that stimulates human β-cell proliferation and insulin secretion, and protects against cytotoxic stress in rat insulinoma cells
Source: PLoS One. 2020 Mar 16;15(3):e0224344. doi: 10.1371/journal.pone.0224344 (PMC7075568; doi:10.1371/journal.pone.0224344)
Supplement: S4 Fig — A preparation of human islets was cultured in the presence of 2.5μM or 5μM GNF-9228 for 72h. and then subjected to the following serial incubation conditions: 1 hour wash, 1 mM glucose; 1 hour incubation, 1 mM glucose; 1 hour incubation 2.5 mM glucose; 1 hour incubation, 16.7 mM glucose. (Data represent mean+Std.Dev. measured in triplicate of 30 islets) (PDF) [file pone.0224344.s004.pdf]

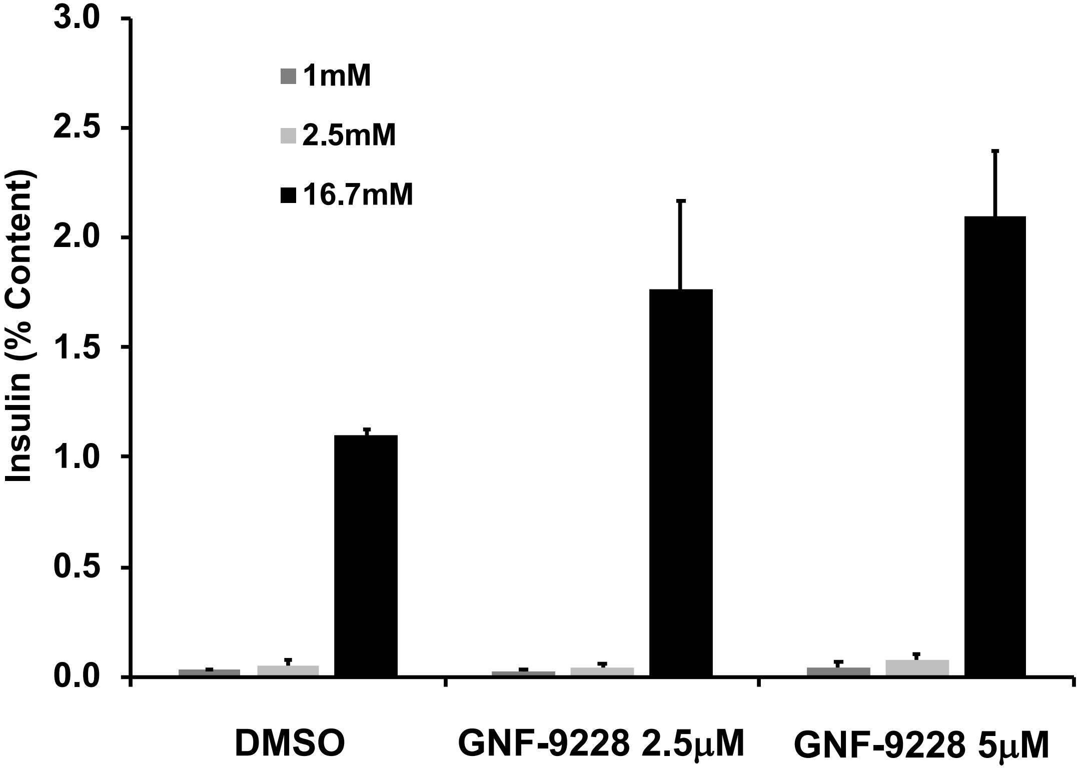

Supplemental Figure 4. Glucose stimulated insulin in human islets at low stimulatory glucose . A preparation of human islets was cultured in the presence of 2.5μM or 5μM GNF-9228 for 72h. and then subjected to the following serial incubation conditions: 1 hour wash, 1 mM glucose; 1 hour incubation, 1 mM glucose; 1 hour incubation 2.5 mM glucose; 1 hour incubation, 16.7 mM glucose. (Data represent mean+ Std.Dev. measured in triplicate of 30 islets)
