# Supplementary material for: Identification of a small molecule that stimulates human β-cell proliferation and insulin secretion, and protects against cytotoxic stress in rat insulinoma cells
Source: PLoS One. 2020 Mar 16;15(3):e0224344. doi: 10.1371/journal.pone.0224344 (PMC7075568; doi:10.1371/journal.pone.0224344)
Supplement: S5 Fig — Rat islets were cultured for 72 h in the presence of 10μM GNF-9228 and 1 μM cyclosporin A (CsA) or DMSO. EdU was added for the last 18 h of culture. Islets were dispersed and stained for EdU incorporation. Immunofluorescent signals were detected and quantified with a Thermo Scientific Cellomics CX5 High Content (HC) cell imaging system. Data are expressed as mean +/- S.E.M. of fold-increase in EdU positive cells in GNF-9228 compared to DMSO-treated rat islets (n = 2 independent rat islet aliquots). (PDF) [file pone.0224344.s005.pdf]

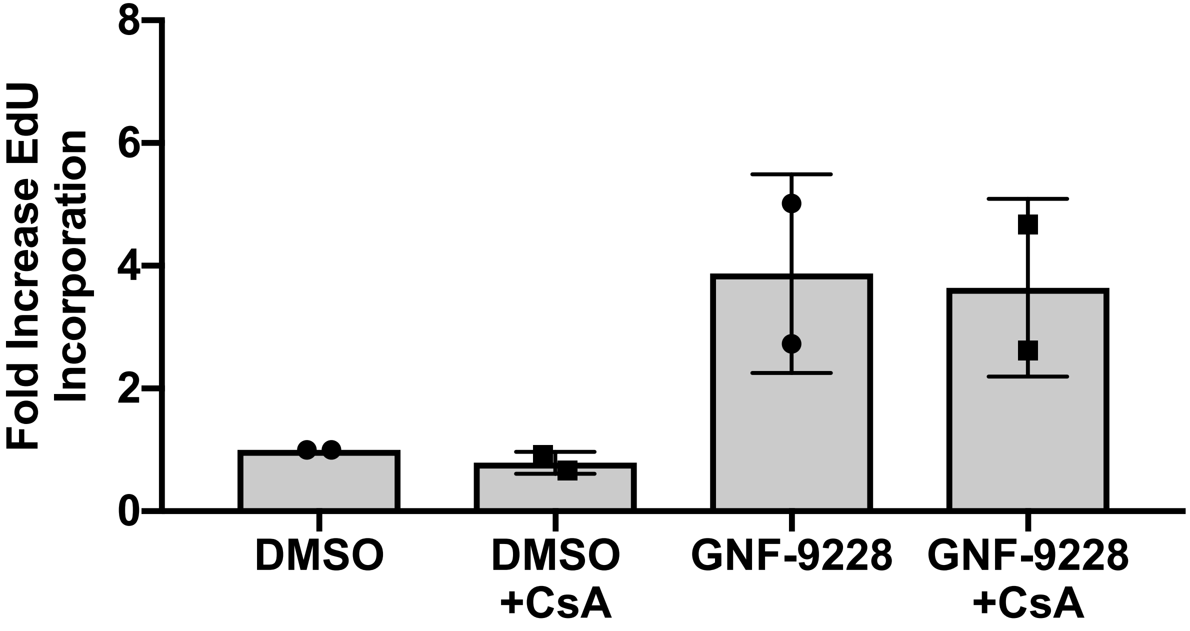

Supplemental Figure 5. Lack of inhibition of GNF-9228-stimulated islet cell EdU incorporation by cyclosporin A in rat islets. Rat islets were cultured for 72 h in the presence of 10μM GNF-9228 and 1 μM cyclosporin A (CsA) or DMSO. EdU was added for the last 18 h of culture. Islets were dispersed and stained for EdU incorporation. Immunofluorescent signals were detected and quantified with a Thermo Scientific Cellomics CX5 High Content (HC) cell imaging system. Data are expressed as mean +/- S.E.M. of fold-increase in EdU positive cells in GNF-9228 compared to DMSO-treated rat islets (n = 2 independent rat islet aliquots).
