# Supplementary material for: Identification of a small molecule that stimulates human β-cell proliferation and insulin secretion, and protects against cytotoxic stress in rat insulinoma cells
Source: PLoS One. 2020 Mar 16;15(3):e0224344. doi: 10.1371/journal.pone.0224344 (PMC7075568; doi:10.1371/journal.pone.0224344)
Supplement: S6 Fig — Mice received a single intraperitoneal (IP) injection of 30 mg/kg GNF-9228 suspended in DMSO, and levels of the compound were measured in blood samples collected at the indicated intervals after injection. Blood was sampled from 2–3 mice at each time point. (PDF) [file pone.0224344.s006.pdf]

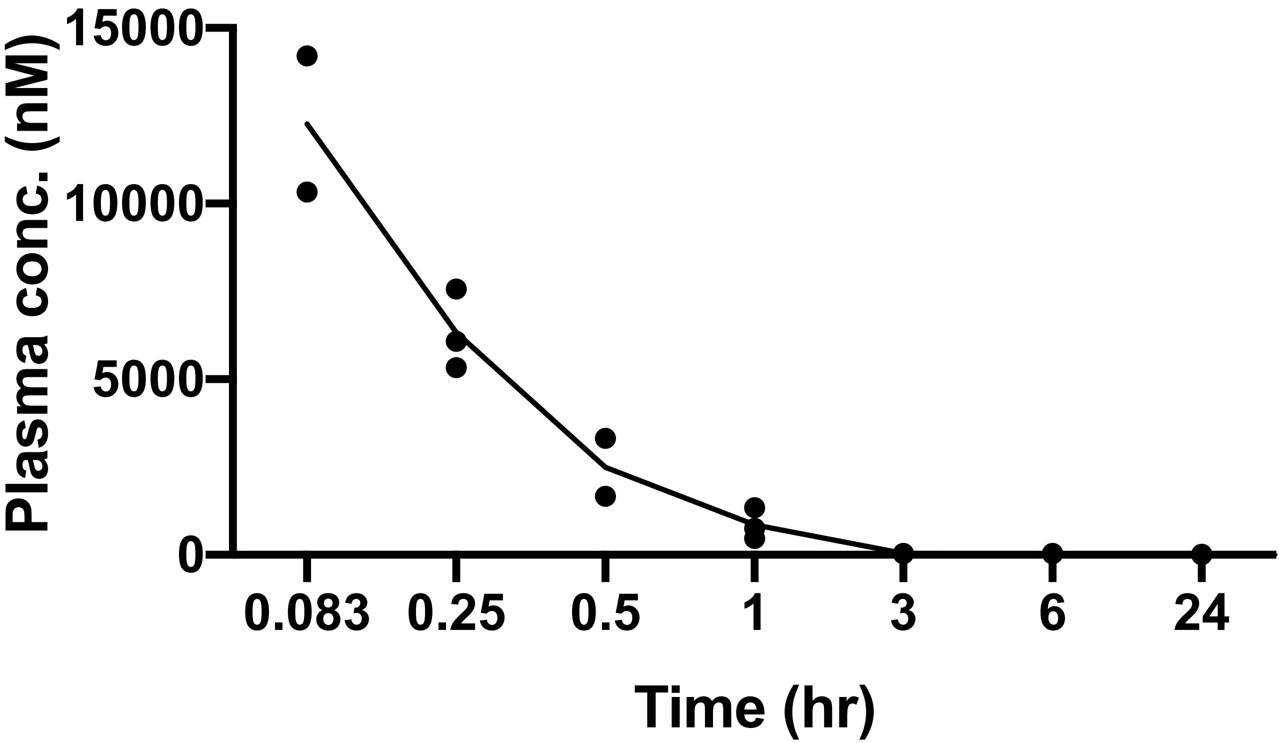

Supplemental Figure 6. Rapid clearance of GNF-9228 in mice. Mice received a single intraperitoneal (IP) injection of 30 mg/kg GNF-9228 suspended in DMSO, and levels of the compound were measured in blood samples collected at the indicated intervals after injection. Blood was sampled from 2-3 mice at each time point.
