# Supplementary material for: Identification of a small molecule that stimulates human β-cell proliferation and insulin secretion, and protects against cytotoxic stress in rat insulinoma cells
Source: PLoS One. 2020 Mar 16;15(3):e0224344. doi: 10.1371/journal.pone.0224344 (PMC7075568; doi:10.1371/journal.pone.0224344)
Supplement: S1 Table — The number of cells assayed and the total percent of Edu positive cells (Edu%), Edu + insulin positive cells (Edu/Ins%), and Edu + glucagon positive cells (EdU/gcg%) for the 7 independent human islet preps summarized in Fig 3 are shown. (PDF) [file pone.0224344.s007.pdf]

Supplemental Table 1. Human islet EdU incorporation studies. The number of cells assayed and the total percent of Edu positive cells (Edu%), Edu + insulin positive cells (Edu/Ins%), and Edu + glucagon positive cells (EdU/gcg%) for the 7 independent human islet preps summarized in Figure 3 are shown.

Cell number

	DMSO	GNF- 9228
Exp.270	not recorded	not recorded
Exp.333	10650	9641
Exp.334A	1707	1558
Exp.334B	2797	2569
Exp.533	4453	3204
Exp.538	10295	7857
Exp.543	6191	7758

Edu%

	DMSO	GNF-9228
Exp.270	0.1135	1.3195
Exp.333	0.17	2.064
Exp.334A	1.113	1.926
Exp.334B	0.429	0.817
Exp.533	0.045	0.156
Exp.538	0.078	0.573
Exp.543	0.032	0.129

EdU / Ins %

	DMSO	GNF-9228
Exp.270	0.059	0.866
Exp.333	0.259	2.683
Exp.334A	1.386	2.54
Exp.334B	0.773	1.294
Exp.533	0.053	0.14
Exp.538	0.076	0.592
Exp.543	0.052	0.66

EdU / gcg %

	DMSO	GNF-9228
Exp.270	0.057	0.31
Exp.333	0.0485	0.4305
Exp.334A	1.79	2.392
Exp.334B	0.427	1.034
Exp.533	0	0.523
Exp.538	0.22	0.844
Exp.543	0	0.149
