# Supplementary material for: Identification of a small molecule that stimulates human β-cell proliferation and insulin secretion, and protects against cytotoxic stress in rat insulinoma cells
Source: PLoS One. 2020 Mar 16;15(3):e0224344. doi: 10.1371/journal.pone.0224344 (PMC7075568; doi:10.1371/journal.pone.0224344)
Supplement: S2 Table — The number of cells assayed and the total percent of Edu positive cells (Edu%), Edu + insulin positive cells (Edu/Ins%), and Edu + glucagon positive cells (EdU/gcg%) for the 6 independent human islet preps summarized in Fig 7 are shown. (PDF) [file pone.0224344.s008.pdf]

Supplemental Table 2. Human islet Edu incorporation studies, in support of Figure 7. The number of cells assayed and the total percent of Edu positive cells (Edu%), Edu + insulin positive cells (Edu/Ins%), and Edu + glucagon positive cells (EdU/gcg%) for the 6 independent human islet preps summarized in Figure 7 are shown.

Islet Cells in Analysis

	DMSO	GNF-9228	GNF-4877	GNF9228+ GNF-4877
Exp.370	7045	5728	8235	9787
Exp.453	12087	15311	13508	8077
Exp.460	5183	8856	8374	7988
Exp.533	15324	10950	8321	10748
Exp.538	19139	11947	23555	20319
Exp.543	17494	33837	38219	25298

EdU %

	DMSO	GNF-9228	GNF-4877	GNF9228+ GNF-4877
Exp.370	0.365	4.7145	0.8085	5.682
Exp.453	0.1915	1.8755	0.7945	2.1585
Exp.460	0.0295	0.6525	0.3825	0.762
Exp.533	0.0855	0.337	0.3595	0.311
Exp.538	0.2155	0.81	0.811	0.8075
Exp.543	0.017	0.124	0.057	0.154

Ins / EdU %

	DMSO	GNF-9228	GNF-4877	GNF9228+ GNF-4877
Exp.370	0.221	3.403	0.544	4.608
Exp.453	0.268	1.782	0.71	1.783
Exp.460	0.026	0.571	0.272	0.485
Exp.533	0.111	0.398	0.348	0.387
Exp.538	0.222	0.807	0.783	0.809
Exp.543	0.127	0.55	0.2535	0.9625

gcg / EdU %

	DMSO	GNF-9228	GNF-4877	GNF9228+ GNF-4877
Exp.370	0.094	0.629	0.073	0.64
Exp.453	0.131	0.621	0.208	0.503
Exp.460	0	0.144	0.049	0.354
Exp.533	0.169	0.854	0.672	0.765
Exp.538	0.404	2.067	2.032	2.247
Exp.543	0.0395	0.1415	0.1345	0.2095
