# Supplementary material for: Identification of a small molecule that stimulates human β-cell proliferation and insulin secretion, and protects against cytotoxic stress in rat insulinoma cells
Source: PLoS One. 2020 Mar 16;15(3):e0224344. doi: 10.1371/journal.pone.0224344 (PMC7075568; doi:10.1371/journal.pone.0224344)
Supplement: S3 Table — The number of cells assayed and the percent of Edu positive + somatostatin positive cells (Edu/sst%) for 3 human islet preps exposed to EdU for 18 h, and 2 human islet preps exposed to EdU for 72 h are shown. (PDF) [file pone.0224344.s009.pdf]

Supplemental Table 3. Human islet Edu incorporation studies in somatostatin positive cells. The number of cells assayed and the percent of Edu positive + somatostatin positive cells (Edu/sst%) for 3 human islet preps exposed to EdU for 18 h, and 2 human islet preps exposed to EdU for 72 h are shown.

Percent Human δ-cell proliferation(18h EdU)

Cell number

	DMSO	GNF- 9228
Exp.533	5000	3814
Exp.538	11637	8009
Exp.543	7774	9167

sst / Edu%

	DMSO	GNF-9228
Exp.533	0.565	0
Exp.538	0	0.353
Exp.543	6	5.455

Percent Human δ-cell proliferation(72h EdU)

Islet Cells in Analysis

	DMSO	GNF-9228	GNF-4877	GNF9228+ GNF-4877
Exp.533	7041	5849	4628	5542
Exp.538	10113	4677	9919	8779

sst / EdU %

	DMSO	GNF-9228	GNF-4877	GNF9228+ GNF-4877
Exp.533	0.488	0.648	0	0.495
Exp.538	0	0	2.007	0.794
